# Molecular‐Level Insights Into Alkali‐Metal‐Cation–CO_2_ Interactions at Electrified Cu(100) Interfaces by Molecular Dynamics Simulations

**DOI:** 10.1002/cssc.71037

**Published:** 2026-09-06

**Authors:** Asato Inoue, Kazuhide Kamiya

**Affiliations:** ^1^ Research Center for Solar Energy Chemistry, Graduate School of Engineering Science, The University of Osaka Toyonaka Osaka Japan; ^2^ Innovative Catalysis Science Division, Institute for Open and Transdisciplinary Research Initiatives (ICS‐OTRI), The University of Osaka Suita Osaka Japan

**Keywords:** alkali‐metal‐cation, CO_2_ reduction reaction, dehydration, electric double layer, molecular dynamics simulations

## Abstract

The activity of the electrochemical CO_2_ reduction reaction depends on the electric double layer, where partially dehydrated alkali metal (AM^+^) ions can stabilize CO_2_ molecules and intermediates. However, whether such interaction‐enabled configurations form under electrochemical conditions and how potential controls their probability remain unclear. Herein, we used classical molecular dynamics simulations to evaluate how the hydration environment of AM^+^ ions and their spatial relationship with CO_2_ molecules evolve as functions of the applied potential and alkali‐metal‐cation identity. The simulations revealed that at potentials more negative than −0.92 V versus point of zero charge, larger AM^+^ ions such as Cs^+^ and K^+^ partially dehydrate near the Cu(100) surface. For K^+^ at *q* = −14.3 μC cm^−2^, the hydration number decreased from 7.5 in the bulk region to 5.6 in the first interfacial layer, while the coordination number of K^+^ around CO_2_ was 0.49 near the surface and 0.20 in the bulk region. The likelihood of partially dehydrated AM^+^ ions residing within the first coordination shell of CO_2_ molecules increased with AM^+^ ion size. At *q* = −9.52 μC cm^−2^, the coordination number of AM^+^ ions around CO_2_ increased from 0.08 for Li^+^ to 0.23 for Cs^+^.

## Graphical abstract

Molecular dynamics simulations reveal how alkali‐metal‐cation identity and applied potential shape the electric double layer at Cu(100) interfaces. Larger cations and more negative potentials promote cation dehydration and direct cation–CO_2_ contact, providing molecular insight into cation‐assisted CO_2_ electroreduction.

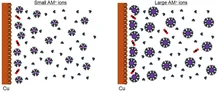

## 1 Introduction

In recent years, global warming and the resultant increase in extreme weather disasters have emerged as critical global challenges, intensifying worldwide efforts toward carbon neutrality [1, 2, 3, 4]. Among various strategies, the electrochemical carbon dioxide reduction reaction (CO_2_RR) has attracted significant attention as a promising on‐site and on‐demand technology for converting anthropogenic CO_2_ into valuable chemicals under ambient temperature and pressure, driven by renewable energy [5, 6, 7]. Among the wide variety of catalysts investigated, Cu‐based materials were uniquely identified in the 1980s as being capable of producing value‐added multicarbon (C_2+_) products such as ethylene, ethanol, acetic acid, and 1‐propanol at appreciable rates [8, 9, 10]. Since then, extensive efforts have been devoted to developing Cu‐based catalysts that enable the CO_2_RR to proceed with superior activity and selectivity [11, 12, 13, 14, 15, 16].

In addition to the molecular structure of the electrocatalyst, the reaction interfaces play a crucial role in determining the activity and selectivity of the CO_2_RR [17, 18, 19, 20]. The region where CO_2_ molecules are reduced and begin to reorganize their molecular structure lies within the electric double layer (EDL). The structure of the EDL and CO_2_RR activities is influenced not only by the atomic and electronic structure of the catalyst surface but also by factors such as the type and concentration of electrolyte ions and solvents.

Among these factors, the electrolyte, particularly the identity of the alkali metal ion (AM^+^ ion), plays a pivotal role in defining the structure of the reaction interface and, consequently, in governing the CO_2_RR activity. Monteiro et al. experimentally demonstrated that the CO_2_RR does not proceed in the absence of AM^+^ ions [21]. In addition, their ab initio molecular dynamics (AIMD) simulations revealed that partially dehydrated AM^+^ ions directly interact with CO_2_ molecules, thereby stabilizing the formation of the CO_2_^−^ intermediate, which is the first elementary step for the CO_2_RR. Zhang et al. further investigated alkali‐metal‐cation effects on CO_2_ activation and the competing hydrogen evolution reaction on Cu(100) using AIMD simulations with enhanced sampling. They showed that the CO_2_ activation barrier decreases from Li^+^ to Cs^+^ because larger cations coordinate more effectively with adsorbed *CO_2_ [22]. Liu et al. investigated the influence of AM^+^ ions on the CO dimerization reaction using AIMD simulations with the Blue Moon ensemble method and revealed that larger AM^+^ ions facilitate CO dimerization by simultaneously interacting with two CO molecules [23]. Through such angstrom‐to‐nanometer‐scale studies, the mechanistic roles of AM^+^ ions in facilitating the CO_2_RR have become increasingly well understood [24, 25, 26, 27].

However, although these AIMD studies have clarified local cation–intermediate interactions and elementary‐step energetics, their inherent spatial and temporal limitations imposed by computational cost prevent them from capturing the full structure of the EDL or the timescales over which the actual reactions proceed. During the CO_2_RR, at the electrode–electrolyte interface where the reaction occurs, various molecules and ions exhibit orientations and arrangements that differ markedly from those in the bulk electrolyte. The initial intermediate in the CO_2_RR, CO_2_^−^, and the key intermediates involved in C_2+_ formation have been reported to be stabilized through direct interactions with bare AM^+^ ions, as described above [21]. However, how often such cations and adsorbed species actually coexist in configurations that enable these interactions or how their likelihood evolves with the applied potential and cation identity remains poorly understood. A further open question is whether AM^+^ ions, which are presumed to remain fully hydrated in the bulk solution, can in fact shed their hydration shell and engage in direct interactions with intermediates at meaningful rates under CO_2_RR conditions. To address these unresolved issues, it is essential to investigate the roles and dynamics of AM^+^ ions within the EDL under CO_2_RR conditions using larger‐scale models that explicitly include molecular representations.

In the present study, we carried out classical molecular dynamics (Classical MD) simulations to investigate the interface between a Cu(100) surface and 1 M (mol L^−1^) AMOH (AM = Li, Na, K, Cs) aqueous solution containing CO_2_ molecules. Classical MD enables simulations extending over tens of nanoseconds and encompassing tens of thousands of atoms, thereby allowing explicit analysis of the configurations and orientations of various molecules within the EDL under different applied potentials [28, 29, 30]. In particular, we investigated the potential‐dependent distributions of various molecules near the Cu surface, focusing on the dehydration behavior of AM^+^ ions and their positional relationship with CO_2_ molecules. The simulations revealed that, under negative potentials, AM^+^ ions form layered concentration profiles near the Cu surface, and their coordination with CO_2_ molecules is markedly enhanced, particularly for larger AM^+^ ions such as Cs^+^ and K^+^.

## 2 Computational Details

**FIGURE 1:**
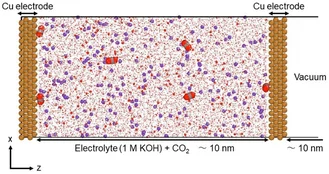
A representative simulation cell containing 1 M KOH solutions and six CO_2_ molecules at *q* = −9.52 μC cm^−2^ on a Cu electrode. Atom colors: C = gray, O = red, H = white, K^+^ = purple, Cu = brown.

Figure 1 presents a representative unit cell used in our MD simulations. Two parallel Cu(100) slabs, each consisting of four atomic layers (dimensions: 52.08 Å × 52.08 Å), were constructed. Solvent, electrolyte, and CO_2_ molecules were placed in the inter‐slab region. The distance between the two Cu slabs was adjusted to approximately 10 nm to keep the density of the electrolyte in the bulk region the same as the bulk electrolyte density calculated at 1 atm and 300 K under the isothermal–isobaric (NPT) ensemble (Table S1). To avoid interactions between the charged Cu surfaces and the counter electrode’s EDL, a vacuum region with a thickness of approximately 10 nm was introduced within the unit cell. The positions of the Cu slabs were fixed, and the Lennard–Jones (LJ) parameters were determined on the basis of the previous report by Naik et al. [31]. The LJ parameters reported by Naik et al. were used for Cu because they were specifically developed for fcc metal surfaces and interfaces and are suitable for describing nonbonded Cu–water/electrolyte interactions in classical MD simulations. The electrolyte solution was composed of 9067 water molecules described with the extended simple point charge model (SPC/E) force field [32] and 163 hydroxide anions parameterized based on the previous report by Netz et al. [33] and AM^+^ ions (Li^+^, Na^+^, K^+^ and Cs^+^), for which force‐field parameters were taken from previous works [30, 34]. The default number of AM^+^ ions was 163; however, this value was adjusted according to the surface charge imposed on the Cu slabs as described below. Six CO_2_ molecules were added, corresponding to the solubility in water, and parameterized using TraPPE [35]. The TraPPE model was used for CO_2_ because it is a well‐established nonreactive force field for describing thermodynamic properties and noncovalent interactions in CO_2_‐containing systems. A summary of all of the force‐field parameters is provided in Table S2. All molecules are considered rigid.

Nonbonding van der Waals interactions were modeled using the 12‐6 LJ potential with a cutoff distance of 12 Å, and the Lorentz–Berthelot mixing rule was adopted to determine the LJ potential parameters between different types of atoms [36]. Long‐range Coulombic interactions beyond 12 Å were evaluated using the particle–particle particle–mesh (PPPM) method. The system temperature was maintained at 300 K using a Nosé–Hoover thermostat [37, 38]. Periodic boundary conditions were applied in all three dimensions. The validity of the force fields was assessed by comparing the first peak positions of $g_{O-H_{2}O\_{\mathrm{AM}}^{+}}(r)$ and the hydration coordination numbers of AM^+^ ions with previously reported experimental and computational values (Table S3). The obtained values were comparable to the reported data, indicating that the present computational setups reasonably reproduce the hydration environments of AM^+^ ions and are suitable for qualitatively discussing AM^+^ dehydration and AM^+^–CO_2_ contact near the electrified Cu interface [39].

To apply electrode potentials, we adopted the fixed‐charge method (FCM), in which constant charges were imposed on the outermost Cu surface layers [28, 29]. Surface charge densities (*q*) of 0, −4.76, −9.52, and −14.3 μC cm^−2^ were assigned to the Cu surface to represent different applied potential conditions. To ensure overall charge neutrality of the simulation cell, the number of AM^+^ ions was adjusted accordingly: 16 additional AM^+^ ions were added for −4.76 μC cm^−2^, 32 for −9.52 μC cm^−2^, and 48 for −14.3 μC cm^−2^.

MD simulations were run in the open‐source large‐scale atomic/molecular massively parallel simulator (LAMMPS) [40]. All simulations were performed in the NVT ensemble at 300 K with a time step of 1 fs for a total duration of 20 ns (Movie S1). The latter 10 ns of each trajectory was used for statistical analysis. Trajectory snapshots were recorded every 1000 steps (1 ps), yielding 10 000 frames in total for analysis. For the atomic density analysis along the direction normal to the Cu slabs, the simulation cell was divided into bins of 0.1 Å, and the average atomic density profiles were analyzed. In the calculation of *ρ*(*z*), each atom was treated as a charge monopole with a fixed partial charge. Here, *ρ*(*z*) denotes the laterally and temporally averaged charge density at position z along the surface‐normal direction. On the basis of the obtained charge density distribution, the electric‐field and electrostatic‐potential profiles were calculated by numerical integration. The electric field at a given position *z*, E(z), was evaluated according to Equation (1), where $\varepsilon_{0}$ is the vacuum permittivity, and $\rho_{t}(z)$ is the total charge density at position *z*. The electrostatic potential Ø(z) was then obtained using Equation (2), which is an integral form of the Poisson equation.

(1) $$E\left(z\right)=\int_{0}^{z}\frac{\rho_{t}\left(z\right)}{\varepsilon_{0}}\;dz$$

(2) $$Ø\left(z\right)=-\int_{0}^{z}E\left(z\right)\;dz$$

Radial distribution function (RDF) analysis was carried out with a bin width of 0.1 Å and a cutoff distance of 20 Å. The RDF for atom type A around atom type B at a distance r from B, $g_{A\_B}(r)$ was calculated using Equation (3), where $n_{A\_B}(r)$ is the local number density of atom type A at a distance *r* from B, and $n_{A}$ is the average number density of atom type A in the entire region confined between the Cu slabs. The coordination number for atom type A around B within a distance *r*, $N_{A\_B}(r)$, was then obtained using Equation (4).

(3) $$g_{\mathrm{A\_B}}\;\left(r\right)\;=\;\frac{n_{A\_B}\;(r)}{4\text{π}r^{2}n_{A}}$$

(4) $$N_{A\_B}\;\left(r\right)=4\text{π}n_{A}\int_{0}^{r}g_{A\_B}\;\left(r\right)\;r^{2}dr$$

## 3 Results and Discussion

### 3.1 Effect of Applied Potential

**FIGURE 2:**
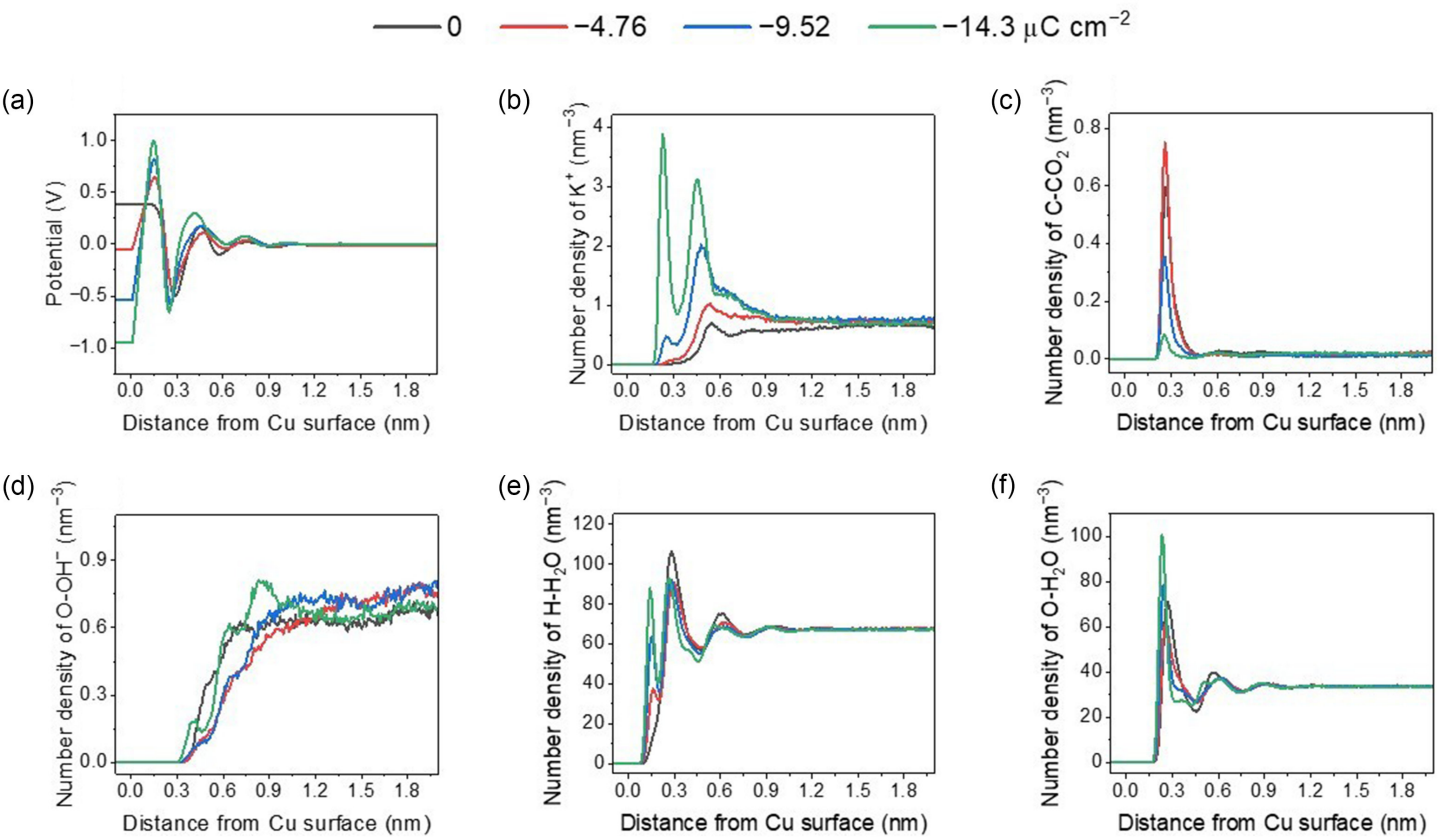
(a) Potential profiles relative to the bulk electrolyte region and number density distributions of (b) K^+^ ions, (c) C‐CO_2_, (d) O‐OH^−^ ions, (e) H‐H_2_O, and (f) O‐H_2_O as a function of the number of charges assigned on the Cu surface.

Figure 2 summarizes the potential profile and spatial distribution profiles for various species as a function of the distance from the Cu surface in the simulation cell containing six CO_2_ molecules dissolved in 1 M KOH solution between two Cu slabs. First, the potential profile relative to the bulk electrolyte region was obtained by numerical integration of the total charge density distribution along the *z*‐axis using Equations (1) and (2) (Figure 2a). The resultant potentials relative to the bulk region were +0.39 V for *q* = 0 μC cm^−2^, −0.05 V for *q* = −4.76 μC cm^−2^, −0.53 V for *q* = −9.52 μC cm^−2^, and −0.95 V for *q* = −14.3 μC cm^−2^. When referenced to the point of zero charge (PZC, *q* = 0 μC cm^−2^) conditions, the corresponding electrode potentials were −0.44, −0.92, and −1.34 V versus PZC for *q* = −4.76, −9.52, and −14.3 μC cm^−2^, respectively. For experimental comparison, the simulated potentials can be approximately converted to the RHE scale by using the reported PZC of Cu(100)/0.1 M NaOH interfaces (−0.17 V vs. RHE) [41]. Therefore, the simulated potentials of −0.44, −0.92, and −1.34 V versus PZC roughly correspond to ca. −0.61, −1.09, and −1.51 V versus RHE, respectively. This conversion is approximate because PZC depends on the surface structure, electrolyte composition, and interfacial adsorbates. Regarding the spatial distribution of K^+^ ions, as the negative charge on the Cu surface increased, K^+^ ions accumulated more strongly near the Cu surface, forming a layered distribution (Figure 2b). Under the *q* = −14.3 μC cm^−2^ condition, the first peak appeared at 2.3 Å from the Cu surface, with a high number density of 3.9 nm^−3^. For C atoms of CO_2_ molecules (C‐CO_2_), the number density of C‐CO_2_ near the Cu surface decreased with increasing charge on the Cu surface (Figure 2c). However, even under the most negative condition (*q* = −14.3 μC cm^−2^), the number density of C‐CO_2_ near the Cu surface (0.08 nm^−3^) remained higher than that in the bulk region (0.02 nm^−3^). The number density of O atoms of OH^−^ ions (O‐OH^−^) showed no substantial change near the Cu surface with increasing negative charge (Figure 2d). For H and O atoms of H_2_O (H‐H_2_O and O‐H_2_O, respectively), the negatively charged surface induced layered distributions (Figure 2e,f). At *q* = −14.3 μC cm^−2^, the number density of O‐H_2_O reached 101 nm^−3^ at 2.3 Å from the Cu surface. These distributions indicate that, under highly negative potentials, H_2_O molecules align with their H atoms oriented toward the Cu surface, forming an ordered interfacial distribution.

**FIGURE 3:**
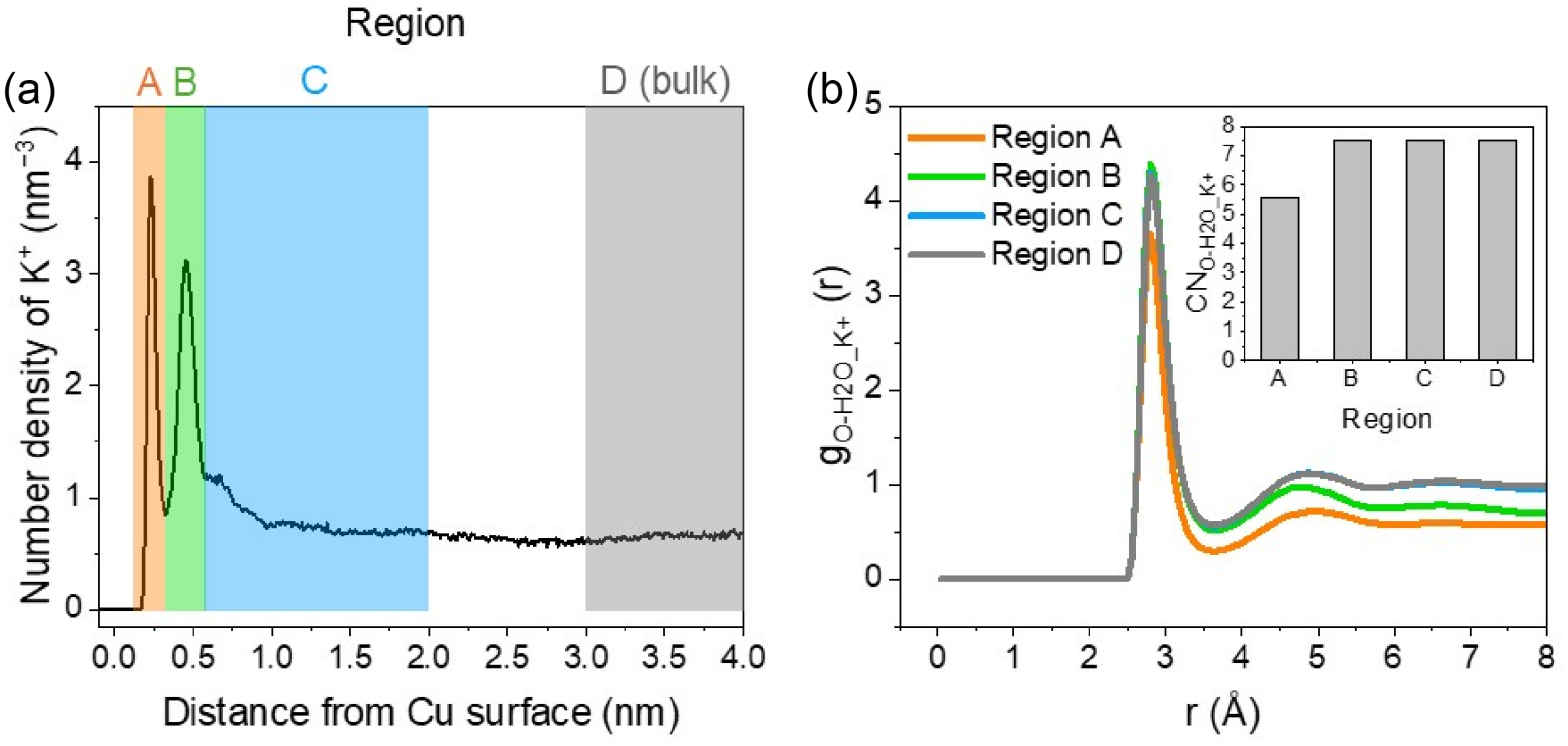
(a) Spatial distribution of K^+^ ions in the vicinity of the Cu surface and (b) RDFs for O‐H_2_O atoms surrounding K^+^ ions ($g_{O-H_{2}O\_K^{+}}(r)$) in Regions A–D under a surface charge of *q* = −14.3 μC cm^−2^. The inset shows the coordination number for O‐H_2_O atoms around K^+^ (${\mathrm{CN}}_{O-H_{2}O\_K^{+}}$) in Regions A–D.

We next discuss the local environment surrounding K^+^ ions near the Cu surface. As described above, increasing the negative surface charges on the Cu surface leads to a layered concentration profile for K^+^ ions. Here, we focus on the local solvation structure of K^+^ ions in each layer. Figure 3a shows the number density distribution of K^+^ ions as a function of the distance from the Cu surface under the *q* = −14.3 μC cm^−2^ condition. The layered distribution was divided into distinct regions: Region A (0–3.2 Å from the Cu surface), Region B (3.3–6.0 Å from the Cu surface), Region C (6.1–20.0 Å from the Cu surface), and Region D (bulk electrolyte region, >30.0 Å from the Cu surface). Figure 3b presents the RDFs for O‐H_2_O atoms around K^+^ ions ($g_{O-H_{2}O\_K^{+}}(r)$) in each region. In all the regions, the RDFs for O‐H_2_O atoms exhibited a maximum at 2.75–2.85 Å from the K^+^ ions; however, the RDF value was notably smaller in Region A. The coordination numbers for O‐H_2_O atoms to K^+^ ions (${\mathrm{CN}}_{O-H_{2}O\_K^{+}}$) in the first shell, obtained by integrating the RDFs up to their first minimum according to Equation (4), are summarized in Figure 3b. The coordination number was 5.6 for Region A, whereas those in Regions B–D were 7.5, indicating that K^+^ ions in Region A exist in a partially dehydrated state, losing approximately two water molecules. This result suggests that K^+^ ions near the negatively charged Cu surface partially shed their hydration shell and directly interact with the Cu surface to stabilize the interfacial environment (Figure S1). Similar analyses performed for *q* = 0, −4.76, and −9.52 μC cm^−2^ are summarized in Figures S2–S4. The partially dehydrated K^+^ ions in Region A appeared only when the surface charge exceeded *q* = −9.52 μC cm^−2^, whereas K^+^ ions in Regions B–D maintained similar hydration structures irrespective of the surface charge density.

**FIGURE 4:**
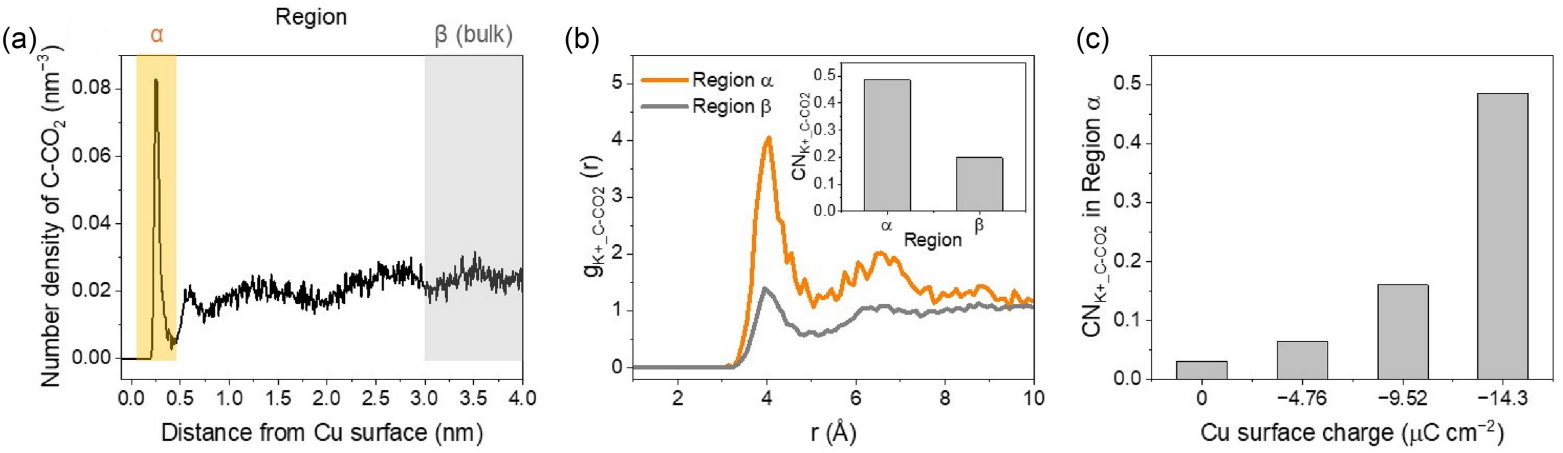
(a) Spatial distribution of C‐CO_2_ atoms in the vicinity of the Cu surface and (b) RDFs for K^+^ ions surrounding C‐CO_2_ ($g_{K^{+}\_C-{\mathrm{CO}}_{2}}(r)$) in Regions α and β under a Cu surface charge of *q* = −14.3 μC cm^−2^. The inset shows coordination number for K^+^ around C‐CO_2_ (${\mathrm{CN}}_{K^{+}\_C-{\mathrm{CO}}_{2}}$) in Regions α and β. (c) ${\mathrm{CN}}_{K^{+}\_C-{\mathrm{CO}}_{2}}$ in Region α under different Cu surface charges.

We here discuss the local environment surrounding CO_2_ molecules near the Cu surface. Figure 4a summarizes the number density profile for C‐CO_2_ atoms under the *q* = −14.3 μC cm^−2^ condition. The number density of C‐CO_2_ reached a peak value of 0.08 nm^−3^ at a distance of 2.5 Å from the Cu surface, which is more than three times the bulk number density (0.02 nm^−3^). The high‐number‐density region was defined as Region α, and the bulk region (>30.0 Å from the Cu surface) was defined as Region β. The RDFs for K^+^ ions around C‐CO_2_ atoms ($g_{K^{+}\_C-{\mathrm{CO}}_{2}}(r)$) in each region are shown in Figure 4b. Both Region α and Region β exhibited an RDF peak at 3.95–4.05 Å from the C‐CO_2_ atoms, indicating that partially dehydrated K^+^ ions are directly coordinated to the CO_2_ molecule without any intervening water molecules. The coordination number for K^+^ ions to C‐CO_2_ atoms (${\mathrm{CN}}_{K^{+}\_C-{\mathrm{CO}}_{2}}$) in the first shell, obtained by integrating the RDFs up to their first minimum according to Equation (4), was 0.49 in Region α and 0.20 in Region β, revealing that K^+^ ions are coordinated to CO_2_ molecules approximately 2.5 times more frequently near the Cu surface compared to in the bulk region (Figure 4b). This behavior is attributed to the higher concentration and enhanced dehydration of K^+^ ions near the negatively charged Cu surface, as discussed in the previous section. Similar analyses for *q* = 0, −4.76, and −9.52 μC cm^−2^ revealed that ${\mathrm{CN}}_{K^{+}\_C-{\mathrm{CO}}_{2}}$ in Region α increases with increasing surface charge on the Cu surfaces (Figure 4c). These results indicate that, as the applied potential becomes more negative, the likelihood that CO_2_ molecules reside near the Cu surface in configurations that enable direct interaction with K^+^ ions increases substantially. To examine the effect of CO_2_ concentration, we performed an additional simulation with a higher CO_2_ concentration of 130 mM, compared with the original concentration of 32.5 mM condition under the *q* = −9.52 μC cm^−2^ condition. Although the C‐CO_2_ number density near the Cu(100) surface increased, the RDF of K^+^ around C‐CO_2_ and the corresponding coordination number were almost unchanged (Figure S5), indicating that the local K^+^‐CO_2_ coordination environment around individual CO_2_ molecules is not strongly affected by CO_2_ concentration.

On the basis of the above analyses, we discuss the potential‐dependent spatial relationship between K^+^ ions and CO_2_ molecules in the context of previously reported experimental and theoretical findings. As described in the Section 1, the influence of K^+^ ions on CO_2_ molecules and CO_2_RR intermediates has been increasingly revealed through recent theoretical investigations. Monteiro et al. reported that direct interactions between partially dehydrated AM^+^ ions and CO_2_^−^ intermediates are indispensable for the progression of the CO_2_RR [21]. In addition, Resasco et al. demonstrated that a strong local electric field generated by cations near the catalyst surface stabilizes the CO_2_^−^ intermediate, thereby promoting the CO_2_RR [25]. Experimentally, applying more negative potentials is widely known to lead to enhanced CO_2_RR activity. For instance, Kuhl et al. showed that, on a Cu electrode in a 0.1‐M KHCO_3_ electrolyte, at −0.75 V versus reversible hydrogen electrode (RHE), the Faradaic efficiencies (FEs) for H_2_ and C_2_H_4_ were 61.8% and 4.3%, respectively; by contrast, at −1.05 V versus RHE, the FE for H_2_ decreased to 22.6%, whereas that for C_2_H_4_ increased to 26.0%, indicating that the CO_2_RR is favored under more negative applied potentials [42].

In the present study, we revealed that an increasingly negative potential substantially enhances the likelihood that CO_2_ molecules reside in close proximity to K^+^ ions in configurations that enable direct interaction. This result demonstrates that the direct interaction between CO_2_ molecules and K^+^ ions—previously proposed by Monteiro et al. [21] as a key factor in CO_2_RR—indeed becomes more prevalent under highly negative applied potentials. In addition, our simulations showed that the local concentration of K^+^ ions near the Cu surface increases with increasing negative applied potential, leading to a stronger interfacial electric field (Figure S6). This amplified electric field is expected to stabilize adsorbed CO_2_^−^ intermediates, as described by Resasco et al. [25] and thus further promote CO_2_RR activity.

Overall, the present simulation demonstrates that the influence of K^+^ ions on CO_2_ electrolysis becomes pronounced at more negative applied potentials. In the next subsection, we systematically explore the dependence of this phenomenon on the identity of the AM^+^ ions to elucidate its generality and mechanistic origin.

### 3.2 Effect of AM^+^ Ion Identity

We now consider the effect of the AM^+^ ion identity on the interfacial structure near the Cu surface. Figure S7 shows the potential profiles and number density distribution for various molecules under the *q* = −9.52 μC cm^−2^ condition for different AM^+^ ions. As shown in Figure S7a, the potential at the Cu surface relative to the bulk electrolyte region was approximately −0.5 V versus bulk, with slight AM^+^‐dependent differences. In the AM^+^ ion number density profiles (Figure S7b), all the AM^+^ ions exhibited a layered distribution. However, for K^+^ and Cs^+^, the first layer appeared within 0.3 nm of the Cu surface, which will be discussed in detail in the following subsection. Regarding the C‐CO_2_ number density shown in Figure S7c, the maximum number density near the Cu surface ranged from 0.35 to 0.53 nm^−3^ for all of the AM^+^ ions, exceeding that in the bulk electrolyte (0.02 nm^−3^). The number density profiles for O‐OH^−^, O‐H_2_O, and H‐H_2_O (Figure S7d–f) showed similar trends irrespective of the type of AM^+^ ions. In particular, the H_2_O molecules exhibited a layered distribution, with H atoms oriented toward the Cu surface, consistent with the results presented in Section 3.1. Larger AM^+^ ions tended to accumulate closer to the Cu surface, consistent with the picture proposed by Resasco et al., in which cation accumulation near the outer Helmholtz plane can modify the interfacial electric field. However, the electric field evaluated using a simple Gauss‐law‐based approach showed only minor AM^+^‐dependent differences near the Cu surface in this study (Figure S8). This is likely because the imposed Cu surface charge density, which was fixed for all AM^+^ species, dominated the electric field in this region. The slight AM^+^‐dependent differences in the potential difference between the bulk electrolyte region and the Cu surface (Figure S7a) can instead be attributed to differences in the ion and charge density distributions slightly away from the Cu surface, particularly at approximately 0.3–0.9 nm. In this region, the AM^+^‐dependent cation distribution is more clearly reflected in the electric‐field profile, and the integration of these small electric‐field differences likely results in the slight AM^+^ dependence of the potential difference.

We next discuss the local environment surrounding AM^+^ ions near the Cu surface under the *q* = −9.52 μC cm^−2^ condition. AM^+^ ions exhibited a layered number density profile along the direction normal to the Cu surface, and only Na^+^, K^+^, and Cs^+^ showed the appearance of a first layer in the region closest to the Cu surface (Region A). The coordination environments for AM^+^ ions in each region were analyzed. Figures S9–S11 show the spatial distribution of AM^+^ ions and the RDFs for O‐H_2_O atoms around AM^+^ ions in the respective regions. The position of O‐H_2_O atoms was found to depend on the ionic radius of each AM^+^ ion, with RDF maxima at 2.35 Å for Li^+^, 2.65 Å for Na^+^, 2.75–2.85 Å for K^+^, and 2.95 Å for Cs^+^. The coordination numbers for O‐H_2_O atoms in the first hydration shell (${\mathrm{CN}}_{O-H_{2}O\_{\mathrm{AM}}^{+}}$), calculated from the RDFs using Equation (4), are summarized in Figure S12. The ${\mathrm{CN}}_{O-H_{2}O\_{\mathrm{AM}}^{+}}$ in the bulk region increased with increasing ionic radius. AM^+^ ions located in Regions B–C exhibited nearly identical ${\mathrm{CN}}_{O-H_{2}O\_{\mathrm{AM}}^{+}}$ as those in the bulk, with values of 5.8 for Li^+^, 6.6–6.7 for Na^+^, 7.5–7.6 for K^+^, and 8.4–8.5 for Cs^+^. Only Na^+^, K^+^, and Cs^+^ were present in Region A, and their ${\mathrm{CN}}_{O-H_{2}O\_{\mathrm{AM}}^{+}}$ values were 5.2 for Na^+^, 5.6 for K^+^, and 6.3 for Cs^+^, corresponding to partial dehydration by approximately two water molecules relative to their bulk hydration structures. Such partially dehydrated AM^+^ ions were observed only for the relatively larger AM^+^ ions (Na^+^, K^+^, and Cs^+^), which is attributable to differences in hydration energies among the AM^+^ ions. Smaller AM^+^ ions such as Li^+^ possess large hydration energies (476 kJ mol^−1^ for Li^+^) [43] and are therefore much less prone to dehydration. By contrast, larger AM^+^ ions such as Na^+^, K^+^, and Cs^+^ have relatively lower hydration energies (371 kJ mol^−1^ for Na^+^, 298 kJ mol^−1^ for K^+^, and 253 kJ mol^−1^ for Cs^+^) [43], allowing partially dehydrated AM^+^ ions to electrostatically interact with the negatively charged Cu surface.

**FIGURE 5:**
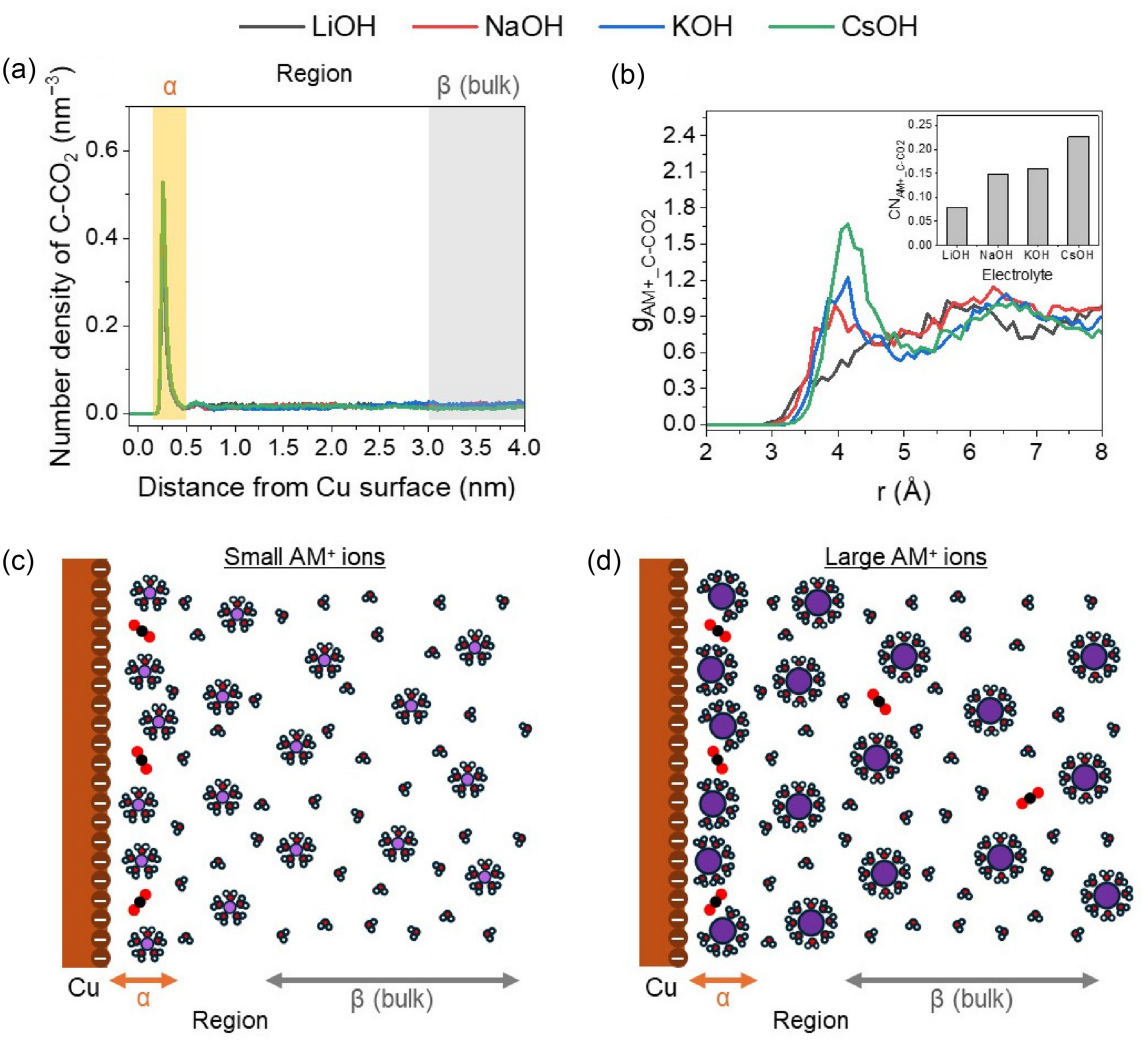
(a) Spatial distribution of C‐CO_2_ atoms in the vicinity of the Cu surface and (b) RDFs for AM^+^ ions surrounding C‐CO_2_ ($g_{{\mathrm{AM}}^{+}\_C-{\mathrm{CO}}_{2}}(r)$) in Region α using different AM^+^ ions under a Cu surface charge of *q* = −9.52 μC cm^−2^. The inset shows the coordination number for AM^+^ ions around C‐CO_2_ (${\mathrm{CN}}_{{\mathrm{AM}}^{+}\_C-{\mathrm{CO}}_{2}}$) in Region α. (c,d) Schematics of the vicinity of the Cu surface depending on the ionic radius of the AM^+^ ions.

Finally, we discuss the local environment of CO_2_ molecules near the Cu surface as a function of the AM^+^ ion species. Figure 5a shows the number density distribution of C‐CO_2_ atoms under the *q* = −9.52 μC cm^−2^ condition for different AM^+^ ions. In all the simulations, the C‐CO_2_ number density becomes higher in Region α, located near the Cu surface, than in the bulk electrolyte (Region β). We subsequently analyzed the relative positions of CO_2_ molecules and AM^+^ ions in Region α. Figure 5b presents the RDFs for AM^+^ ions around C‐CO_2_ atoms ($g_{{\mathrm{AM}}^{+}\_C-{\mathrm{CO}}_{2}}(r)$) in Region α for each AM^+^ ion. The positions of the AM^+^ ions relative to C‐CO_2_ atoms depended on the size of the AM^+^ ions. The first RDF maxima were observed only for Na^+^, K^+^, and Cs^+^, at 3.95, 4.15, and 4.15 Å, respectively, suggesting direct coordination of these ions to CO_2_ molecules without intervening water molecules. In contrast, no distinct RDF maximum was observed for Li^+^. Accordingly, the coordination numbers for AM^+^ ions around C‐CO_2_ atoms $\left({\mathrm{CN}}_{{\mathrm{AM}}^{+}\_C-{\mathrm{CO}}_{2}}\right)$ were calculated using the region up to the first RDF minimum and Equation (4) for Na^+^, K^+^, and Cs^+^, giving values of 0.15, 0.16, and 0.23, respectively, whereas the value for Li^+^ was obtained by integrating the RDF up to 4.55 Å and was found to be 0.08 (Figure 5b). These results demonstrate that the likelihood of AM^+^ ions occupying positions allowing direct, water‐free interactions with CO_2_ molecules increases with the increasing AM^+^ ion size. Figure 5c,d provides schematics of the interfacial environment under *q* = −9.52 μC cm^−2^, highlighting the difference among AM^+^ ions. As discussed earlier, smaller AM^+^ ions such as Li^+^ possess high hydration energies, making dehydration difficult and thereby limiting their ability to interact directly with CO_2_ molecules. By contrast, larger AM^+^ ions such as K^+^ and Cs^+^ have lower hydration energies, allowing them to become partially dehydrated and interact directly with CO_2_ molecules more readily. The present 1 M AMOH plus neutral CO_2_ model should be regarded as an idealized nonreactive model, not an equilibrium speciation model of CO_2_ in an alkaline solution. Although CO_2_ can form bicarbonate and carbonate species in alkaline media, the innermost region near the negatively charged Cu(100) surface in the present simulations was enriched in AM^+^ ions and contained very few OH^−^ anions (Figure S7), suggesting that the AM^+^‐CO_2_ coordination analyzed here mainly reflects a cation‐rich local environment. Quantitative effects of bicarbonate and carbonate species on AM^+^ distributions, local CO_2_ availability, and interfacial electric fields should be examined in future multicomponent or reactive simulations.

On the basis of the above analyses, we discuss the AM^+^‐ion‐species‐dependent spatial relationship between AM^+^ ions and CO_2_ molecules in the context of previously reported experimental findings. Numerous studies have consistently demonstrated that larger AM^+^ ions promote CO_2_RR activity. For example, Singh et al. conducted CO_2_RRs in 0.1 M AMHCO_3_ electrolytes (AM = Li, Na, K, Rb, Cs). In 0.1 M LiHCO_3_, the FEs for CH_4_, C_2_H_4,_ and C_2_H_5_OH were limited to 6.2%, 1.5%, and 0.6%, respectively, whereas H_2_ evolution dominated, with an FE of 85.9% at −1.0 V versus RHE [44]. By contrast, replacing Li^+^ with Cs^+^ substantially promoted the CO_2_RR, resulting in CH_4_, C_2_H_4,_ and C_2_H_5_OH FEs of 9.4%, 31.1%, and 11.4%, respectively, while suppressing H_2_ evolution to 31.1%.

In the present study, we revealed that AM^+^ ions with larger ionic radii and lower hydration energies more readily undergo dehydration and directly interact with CO_2_ molecules, which is an indispensable step for driving the CO_2_RR. This finding elucidates one mechanistic aspect behind the enhanced CO_2_RR performance observed when larger AM^+^ ions are used. Of course, not only the likelihood of such interactions but also the strength of the interactions to stabilize the intermediates is expected to strongly depend on the cation identity. Going forward, integrating quantum chemical calculations with the interfacial EDL structure elucidated in the present study will further refine understanding of the local reaction environment governing CO_2_RR.

## 4 Conclusion

We carried out classical MD simulations to investigate how the local interfacial structure between the Cu(100) surface and an electrolyte with dissolved CO_2_ evolves as a function of the applied potential and the AM^+^ ion identity. The simulations revealed that, under highly negative potentials, AM^+^ ions formed a layered number density distribution in the direction normal to the Cu(100) surface. Notably, when larger AM^+^ ions were used and negative charges of −9.52 μC cm^−2^ or more were applied to the Cu(100) surface, partially dehydrated AM^+^ ions emerged near the Cu(100) surface. In addition, analysis of the local environment surrounding CO_2_ molecules near the Cu(100) surface revealed that the likelihood of partially dehydrated AM^+^ ions occupying the first coordination shell of CO_2_ molecules (i.e., enabling direct interactions with CO_2_ without intervening water molecules) increases with an increasingly negative applied potential and with the use of larger AM^+^ ions. These trends can be rationalized by the smaller hydration energies of larger AM^+^ ions.

Overall, these findings provide molecular‐level insight into one of the mechanistic factors underlying the experimentally observed enhancement of CO_2_RR performance when larger AM^+^ ions or highly negative applied potentials are used. Because the present simulations were limited to Cu(100), the conclusions are directly applicable to Cu(100) interfaces, and other Cu facets may show different interfacial structures and cation distributions. Nevertheless, the present results provide a molecular basis for understanding how cation size and applied potential regulate cation dehydration and cation–CO_2_ contact at a well‐defined Cu(100) interface.

## Funding

This work was supported by the Core Research for Evolutional Science and Technology (JPMJCR24S6) and the Japan Society for the Promotion of Science (23KJ1503 and 23K26756).

## Conflicts of Interest

The authors declare no conflicts of interest.

## Supporting information

Supplementary Material

Supplementary Material

## Data Availability

The data that support the findings of this study are available from the corresponding author upon reasonable request.
