# Supplementary material for: Molecular‐Level Insights Into Alkali‐Metal‐Cation–CO2 Interactions at Electrified Cu(100) Interfaces by Molecular Dynamics Simulations
Source: ChemSusChem. 2026 Sep 6;19(17):e71037. doi: 10.1002/cssc.71037 (PMC13547899; doi:10.1002/cssc.71037)
Supplement: Supplementary file 1 [file CSSC-19-e71037-s001.pdf]

Supporting Information for

Molecular-Level Insights into Alkali-Metal Cation–CO_2 Interactions at Electrified Cu(100) Interfaces by Molecular Dynamics Simulations

Asato Inoue*, and Kazuhide Kamiya*

Table S1. Density of the bulk electrolyte region.

Electrolyte	Density (g/cm^3)
1 M LiOH	1.035
1 M NaOH	1.046
1 M KOH	1.057
1 M CsOH	1.146

Table S2. List of L–J parameters used in this study.

Atom	Mass (g mol^{-1})	Partial charge (e^-)	ε (kcal mol^{-1})	σ (Å)
Cu	63.55	Set by FCM	0.1859	2.34
O (H_2O)	15.9994	−0.8476	0.1553	3.166
H (H_2O)	1.0080	+0.4238	0	0
O (OH^-)	15.9994	−1.0	0.012	3.81
H (OH^-)	1.0080	0	0	0
Li^+	6.941	+1.0	0.1601	2.337
Na^+	22.9898	+1.0	0.1247	2.876
K^+	30.0983	+1.0	0.1247	3.250
Cs^+	132.9055	+1.0	0.1247	3.526
C (CO_2)	12.0107	+0.7	0.0537	2.80
O (CO_2)	15.9994	−0.35	0.157	3.05

Table S3. Comparison of the first peak positions of $g_{\mathrm{O-H2O\_}AM+}(r)$ and the hydration coordination numbers of AM^+ ions obtained in this study with previously reported experimental and computational values.

AM^+	Peak position of $g_{\mathrm{O-H2O\_}AM+}(r)$	Bulk CN	Notes	Reference
Li^+	2.35	5.8	Classical MD	This work
		5.7	Classical MD	S1
		4	Experiment (LAXS)	S2
	1.99	4	Car-Parrinello MD	S3
Na^+	2.65	6.7	Classical MD	This work
		5.9	Classical MD	S1
	2.43	6	Experiment (LAXS)	S2
	2.34	5.3	Experiment (Neutron diffraction)	S4
	2.4	5.2	Car-Parrinello MD	S3
K^+	2.86	7.6	Classical MD	This work
		7.3	Classical MD	S1
	2.81	7	Experiment (LAXS)	S2
	2.65	6	Experiment (Neutron diffraction)	S4
	2.85	7.1	Car-Parrinello MD	S3
Cs^+	2.95	8.5	Classical MD	This work
		10.1	Classical MD	S1
	3.07	8	Experiment (LAXS)	S2

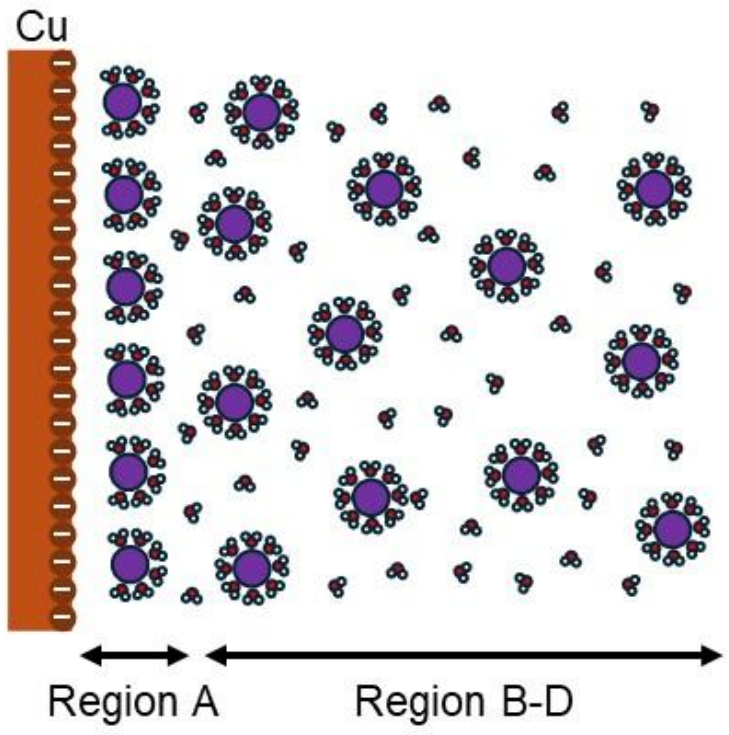

Figure S1. Schematic of the microenvironment focusing on K^+ ions near the Cu surface under a Cu surface charge of $q = -14.3$ μC cm^{-2}.

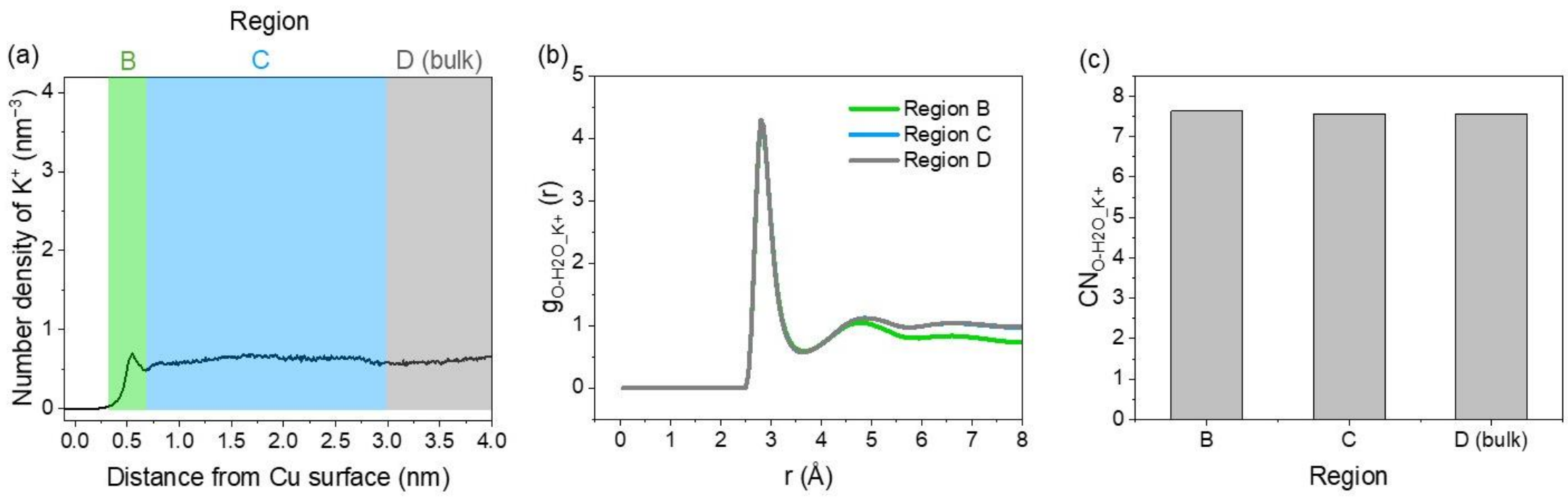

Figure S2. (a) Spatial distribution of K^+ ions near the Cu surface, (b) RDFs for O-H_2O atoms surrounding K^+ ions ($g_{\mathrm{O-H2O\_K+}}(r)$) in Regions B–D and (c) coordination number for O-H_2O atoms around K^+ (CN_{O-H2O_K+}) in Regions B–D under a surface charge of $q = 0$ μC cm^{-2}.

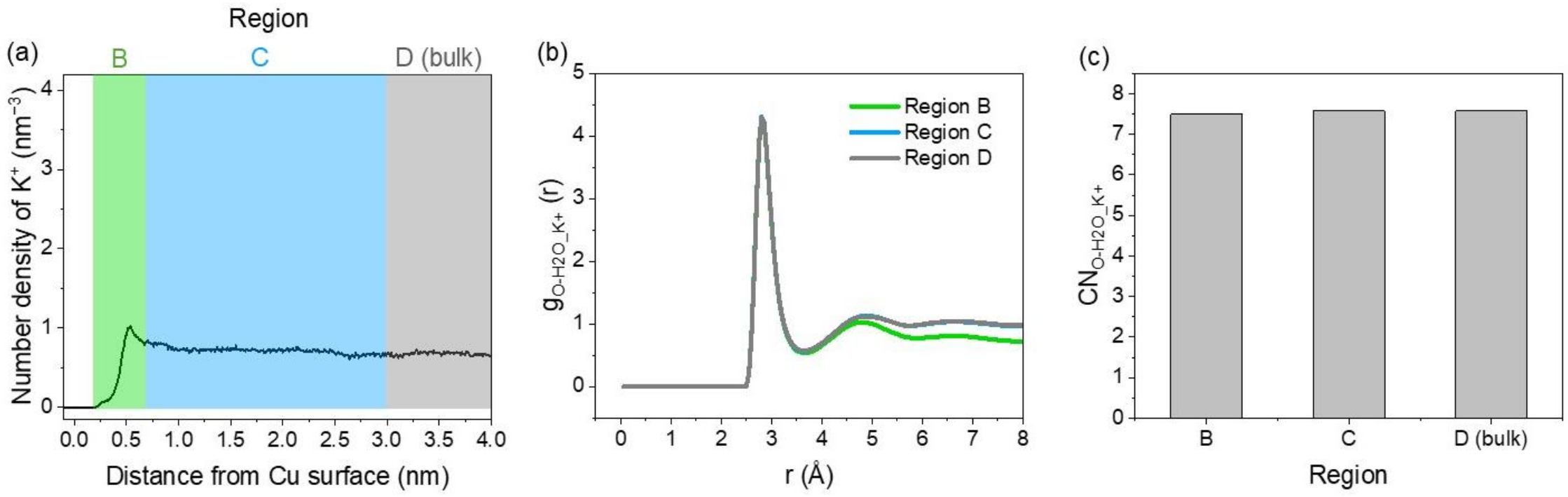

Figure S3. (a) Spatial distribution of K^+ ions near the Cu surface, (b) RDFs for O-H_2O atoms surrounding K^+ ions ($g_{\mathrm{O-H2O\_K+}}(r)$) in Regions B–D and (c) coordination number for O-H_2O atoms around K^+ ($CN_{\mathrm{O-H2O\_K+}}$) in Regions B–D under a surface charge of q = −4.76 μC cm^{-2}.

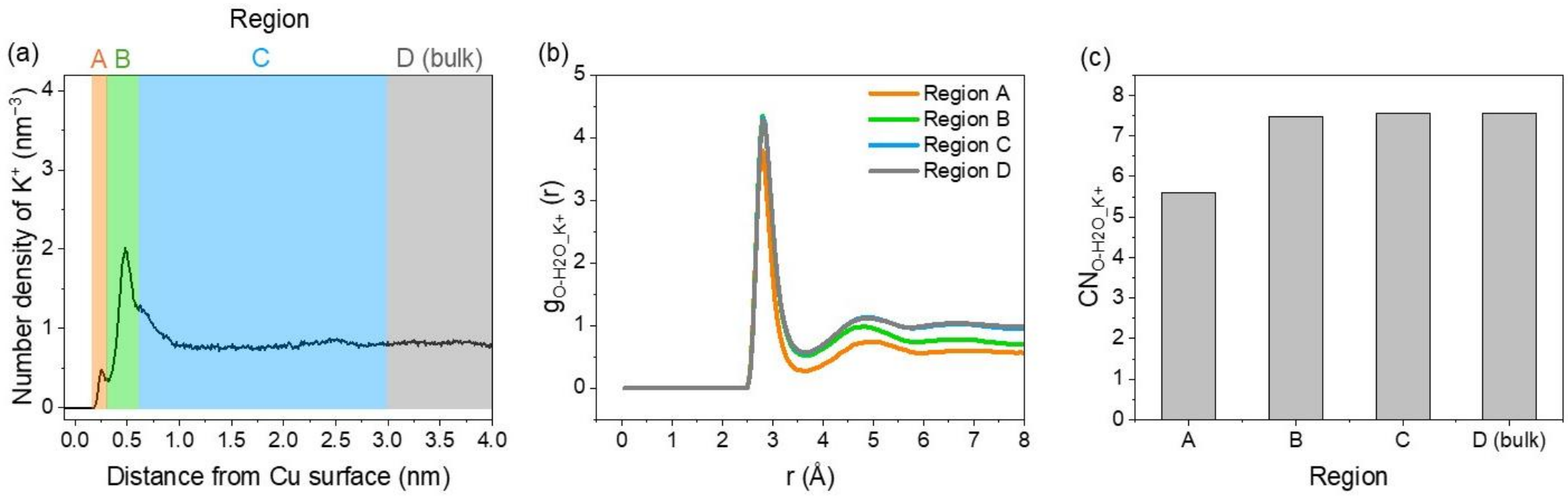

Figure S4. (a) Spatial distribution of K^+ ions near the Cu surface, (b) RDFs for O-H_2O atoms surrounding K^+ ions ($g_{\mathrm{O-H2O\_K+}}(r)$) in Regions A–D and (c) coordination number for O-H_2O atoms around K^+ ($CN_{\mathrm{O-H2O\_K+}}$) in Regions A–D under a surface charge of $q = -9.52$ μC cm^{-2}.

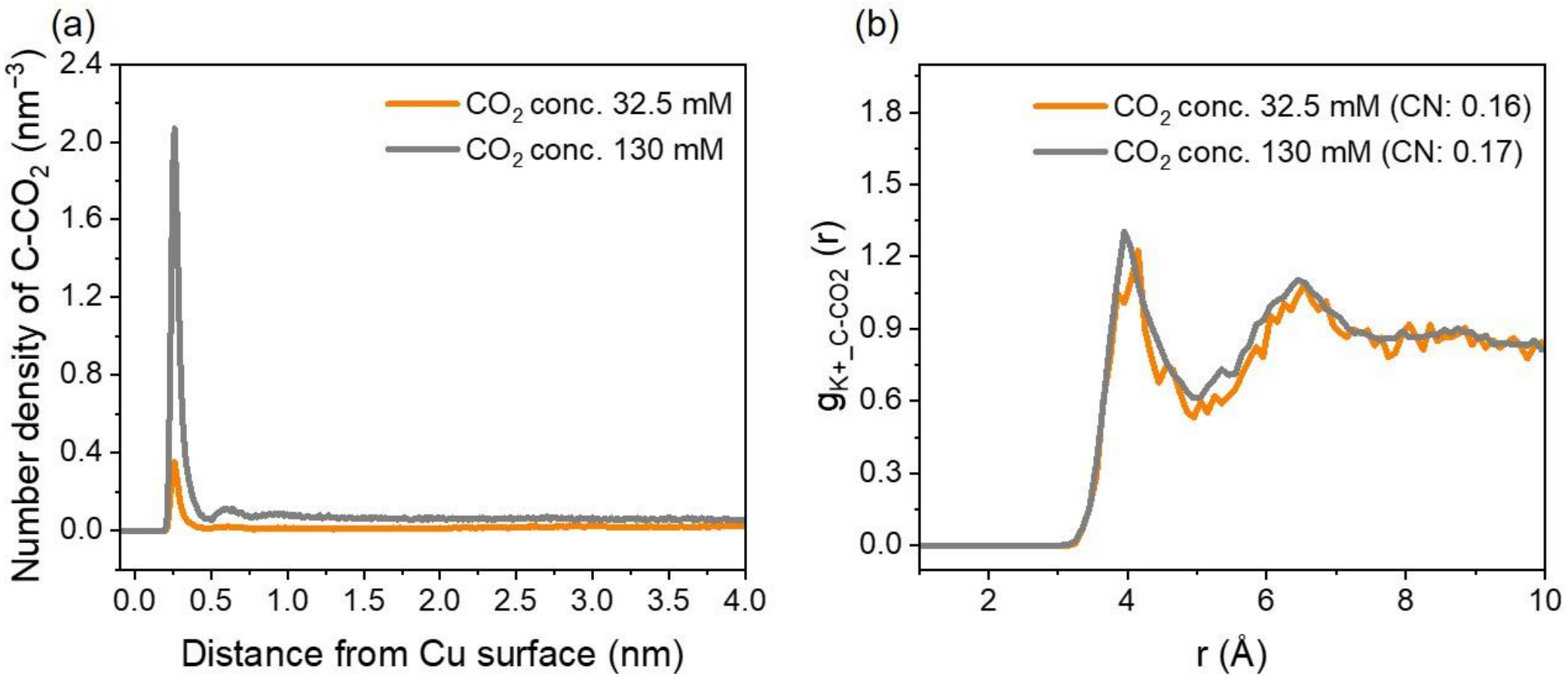

Figure S5. (a) Number density profiles of C-CO_2 near the Cu surface and (b) RDFs for K^+ ions surrounding C-CO_2 ($g_{K+\_C-CO2}(r)$) near the Cu surface under different CO_2 concentrations under a Cu surface charge of $q = -9.52$ μC cm^{-2}.

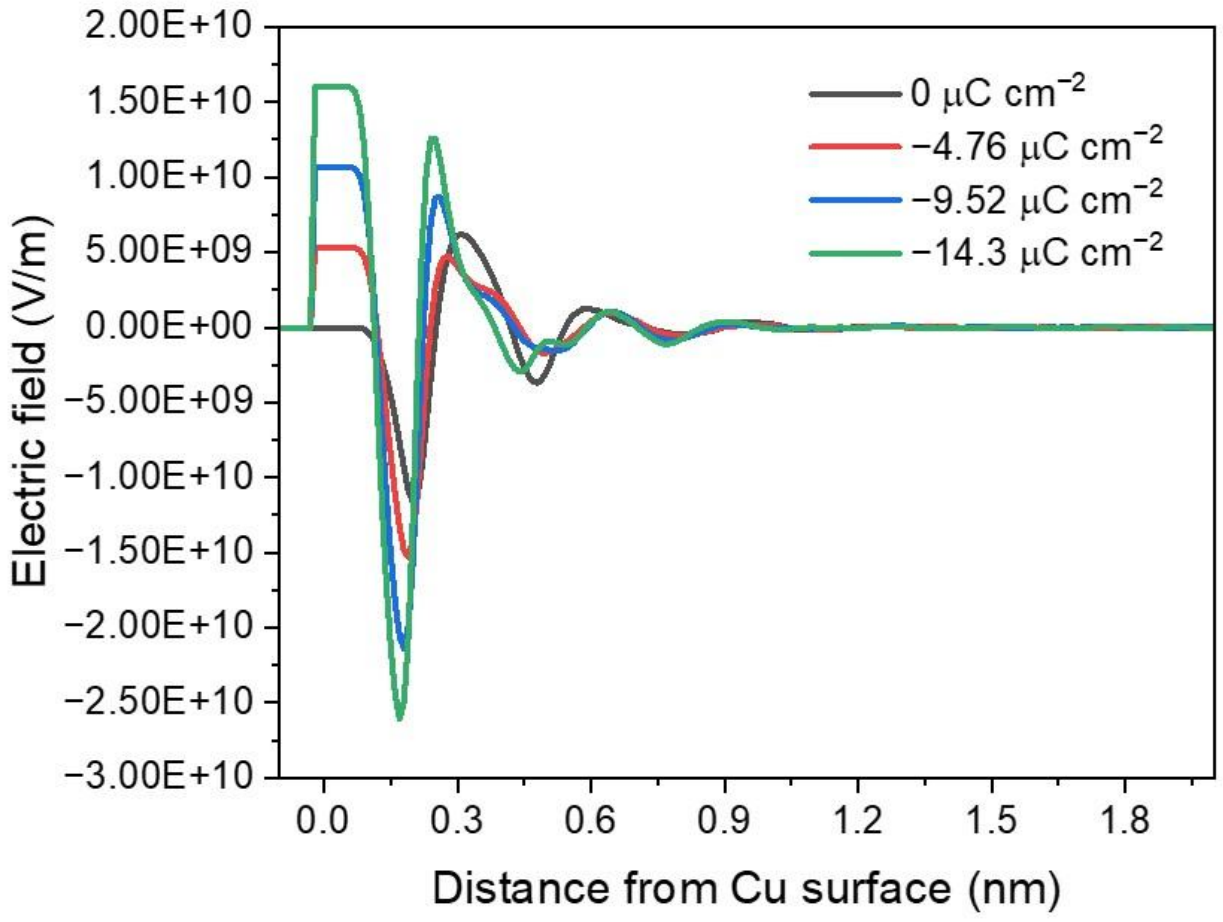

Figure S6. Electric field distribution as a function of the Cu surface charge.

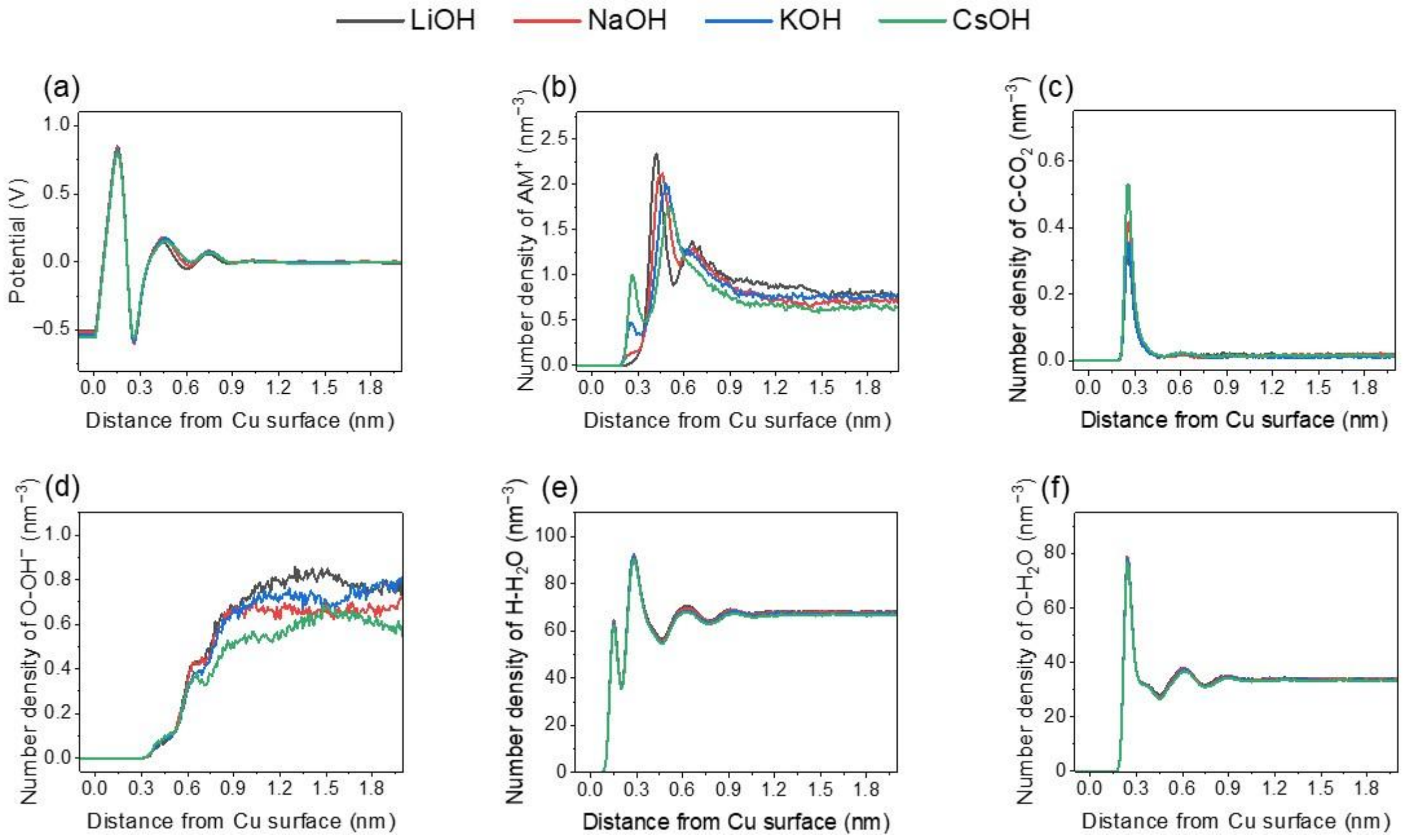

Figure S7. (a) Potential profiles relative to the bulk electrolyte region and number density distributions for (b) AM^+ ions, (c) C-CO_2, (d) O-OH^- ions, (e) H-H_2O and (f) O-H_2O for different AM^+ ions under a surface charge of $q = -9.52$ μC cm^{-2}.

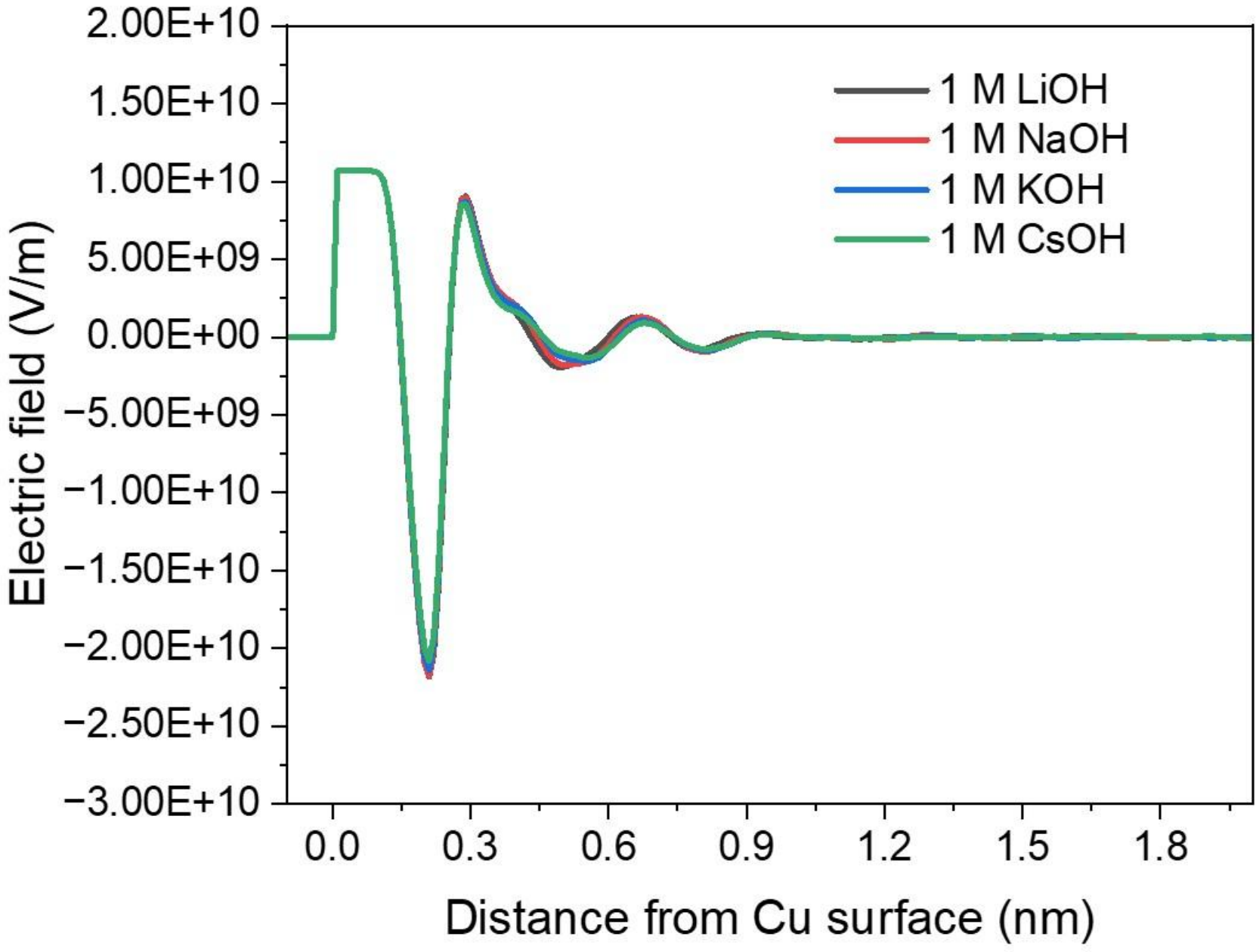

Figure S8. Electric field profiles near the Cu surface for different AM^+ ions under a surface charge density of $q = -9.52$ μC cm^{-2}.

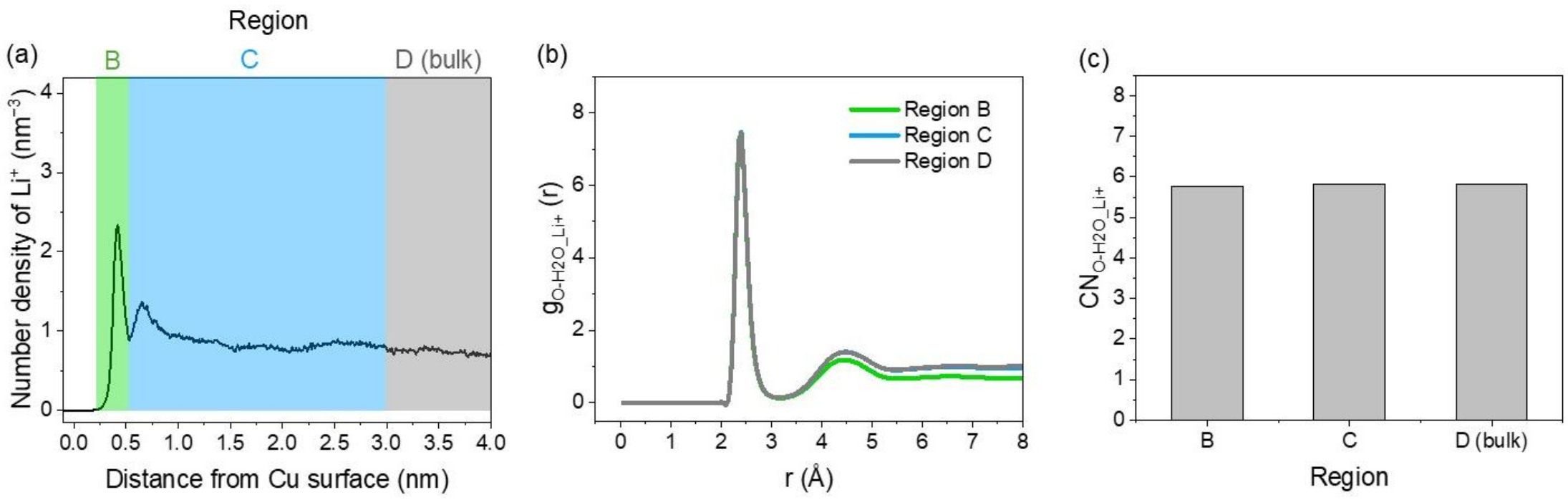

Figure S9. (a) Spatial distribution of Li^+ ions near the Cu surface, (b) RDFs for O-H_2O atoms surrounding Li^+ ions ($g_{\mathrm{O-H2O\_Li+}}(r)$) in Regions B–D and (c) coordination number for O-H_2O atoms around Li^+ ($CN_{\mathrm{O-H2O\_Li+}}$) in Regions B–D under a surface charge of $q = -9.52$ μC cm^{-2}.

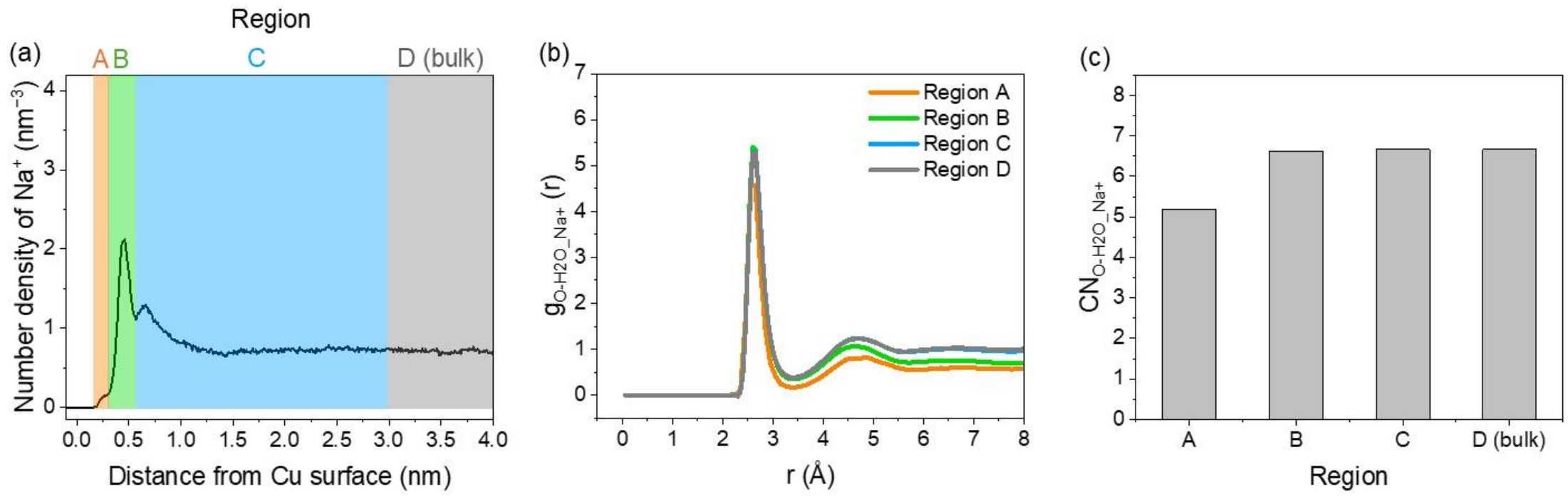

Figure S10. (a) Spatial distribution of Na^+ ions in the vicinity of the Cu surface, (b) RDFs for O-H_2O atoms surrounding Na^+ ions ($g_{\mathrm{O-H2O\_Na+}}(r)$) in Regions A–D and (c) coordination number for O-H_2O atoms around Na^+ ($CN_{\mathrm{O-H2O\_Na+}}$) in Regions A–D under a surface charge of $q = -9.52$ μC cm^{-2}.

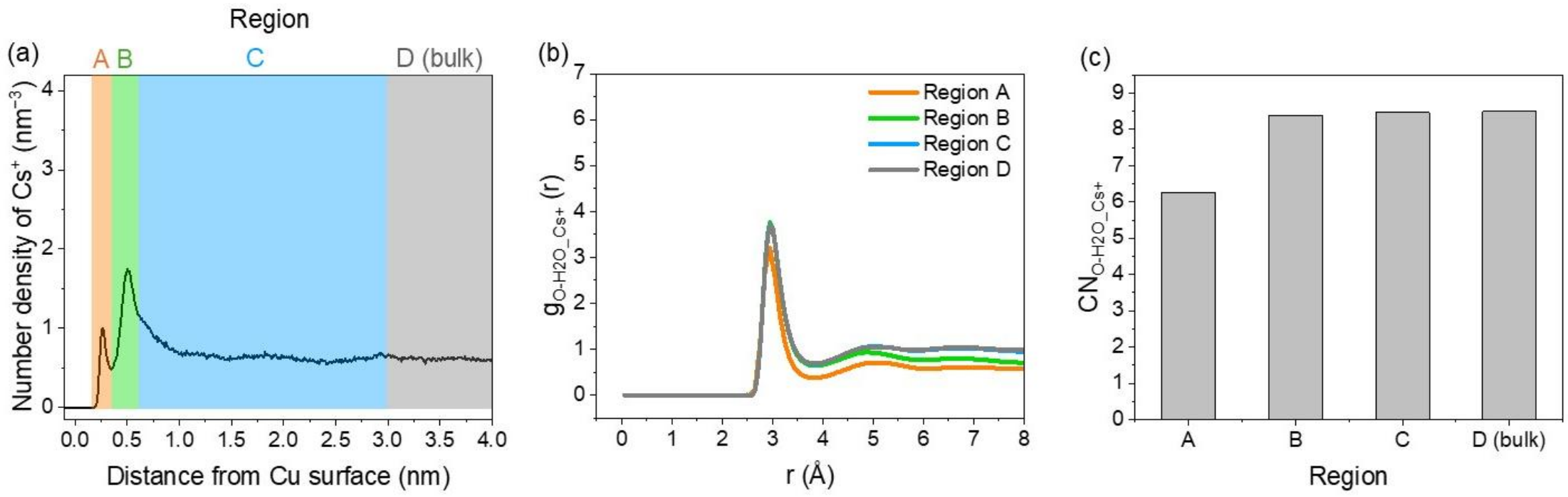

Figure S11. (a) Spatial distribution of Cs^+ ions near the Cu surface, (b) RDFs for O-H_2O atoms surrounding Cs^+ ions ($g_{\mathrm{O-H2O\_Cs+}}(r)$) in Regions A–D and (c) coordination number for O-H_2O atoms around Cs^+ ions ($CN_{\mathrm{O-H2O\_Cs+}}$) in Regions A–D under a surface charge of $q = -9.52$ μC cm^{-2}.

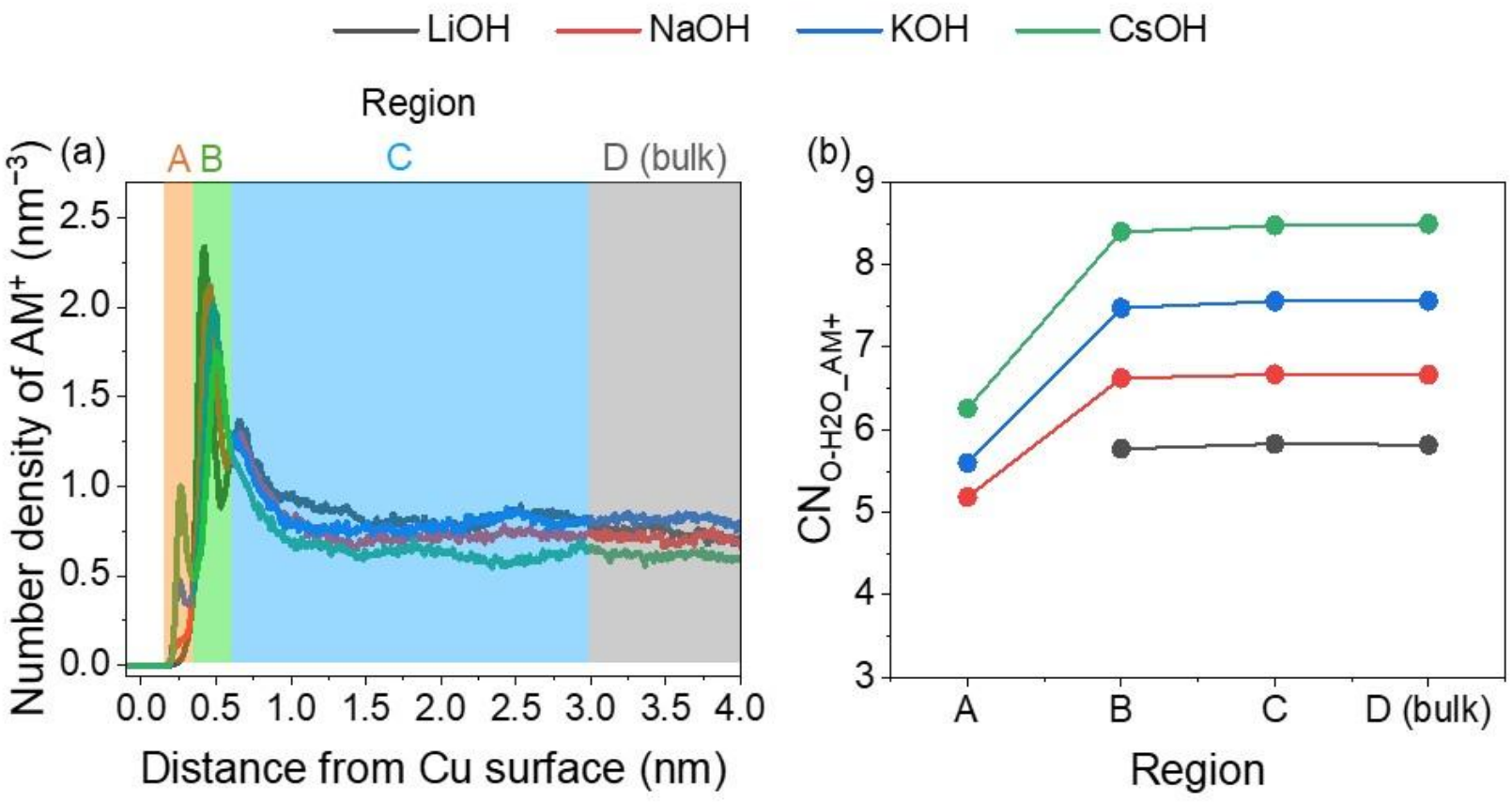

Figure S12. A summary of (a) the spatial distribution of AM^+ ions and (b) coordination number for O-H_2O atoms around AM^+ ions ($CN_{\mathrm{O-H2O\_AM+}}$) in Regions A–D under a surface charge of $q = -9.52$ μC cm^{-2}.

Movie S1. A representative MD trajectory of a simulation cell containing 1 M KOH solutions and six CO_2 molecules at $q = -9.52$ μC cm^{-2} on a Cu electrode. Atom colours: C = gray, O = red, H = white, K^+ = purple, Cu = brown.

References

[S1] E. Johnson, S. Haussener, *J. Phys. Chem. C* **2024***, 128*, 10450–10464.

[S2] J. Mähler, I. Persson, *Inorg. Chem.* **2012**, *51*, 425–438.

[S3] T. Ikeda, M. Boero, K. Terakura, *J. Phys. Chem.* **2007**, *126*, 034501.

[S4] R. Mancinelli, A. Botti, F. Bruni, M. A. Ricci, A. K. Soper, *J. Phys. Chem. B*, **2007**, *111*, 13570–13577.
